# The Positive Inotropic Effect of Pyruvate Involves an Increase in Myofilament Calcium Sensitivity

**DOI:** 10.1371/journal.pone.0063608

**Published:** 2013-05-15

**Authors:** Carlos A. A. Torres, Kenneth D. Varian, Cynthia H. Canan, Jonathan P. Davis, Paul M. L. Janssen

**Affiliations:** Department of Physiology and Cell Biology, College of Medicine, The Ohio State University, Columbus, Ohio, United States of America; Semmelweis University, Hungary

## Abstract

Pyruvate is a metabolic fuel that is a potent inotropic agent. Despite its unique inotropic and antioxidant properties, the molecular mechanism of its inotropic mechanism is still largely unknown. To examine the inotropic effect of pyruvate in parallel with intracellular calcium handling under near physiological conditions, we measured pH, myofilament calcium sensitivity, developed force, and calcium transients in ultra thin rabbit heart trabeculae at 37 °C loaded iontophoretically with the calcium indicator bis-fura-2. By contrasting conditions of control versus sarcoplasmic reticulum block (with either cyclopiazonic acid and ryanodine or with thapsigargin) we were able to characterize and isolate the effects of pyruvate on sarcoplasmic reticulum calcium handling and developed force. A potassium contracture technique was subsequently utilized to assess the force-calcium relationship and thus the myofilament calcium sensitivity. Pyruvate consistently increased developed force whether or not the sarcoplasmic reticulum was blocked (16.8±3.5 to 24.5±5.1 vs. 6.9±2.6 to 12.5±4.4 mN/mm^2^, non-blocked vs. blocked sarcoplasmic reticulum respectively, p<0.001, n = 9). Furthermore, the sensitizing effect of pyruvate on the myofilaments was demonstrated by potassium contractures (EC50 at baseline versus 20 minutes of pyruvate infusion (peak force development) was 701±94 vs. 445±65 nM, p<0.01, n = 6). This study is the first to demonstrate that a leftward shift in myofilament calcium sensitivity is an important mediator of the inotropic effect of pyruvate. This finding can have important implications for future development of therapeutic strategies in the management of heart failure.

## Introduction

Pyruvate is naturally present in circulating blood at concentrations that vary between 0.1 to 0.2 mM [1]. At these levels, pyruvate does not exert any clinically significant enhancement of myocardial contractile strength. However, in concentrations in the range of 3 to 30 mM (which can be achieved via infusion) pyruvate has been shown to improve the contractile strength of the heart up to 200% [2]. The inotropic effect of pyruvate has been demonstrated in both normal and failing hearts. Moreover, it exerts its positive inotropic effects under both hypoxic and post ischemic conditions [3]. Pyruvate’s effects in augmentingthe contractile strength of the heart have been shown to be consistent throughout several species including rat, rabbit, swine, and humans [4], [5], [6], [7]. It potentiates the effect of β-adrenergic drugs [8] and it has been infused intracoronary in humans experimentally to increase inotropic support [4], [9].

Pyruvate has demonstrated additional characteristics that separate it from the current available arsenal of inotropic drugs used for treatment of acute heart failure. Pyruvate is a known antioxidant [10], [11], [12], it is a readily consumable metabolic fuel that has been postulated to enhance glycolysis even during ischemic events [13] and in stark contrast to the currently widely used β-adrenergic agonists, it has recently been shown to not be detrimental to the economy of myocardial contraction [3]. This latter characteristic is of crucial importance at a moment when the myocardium oxygen consumption is critically taxed and its energy reserves are at a premium and can potentially translate into very significant therapeutic advantages.

While the inotropic action of pyruvate is well known, the underlying mechanism is incompletely understood. A review by Mallet [1] suggests among the most probable candidates for the underlying mechanism pyruvate’s enhancement of cytosolic ATP phosphorylation potential and Gibbs free energy of ATP hydrolysis (DGATP) and pyruvate’s effects on the sarcoplasmic reticulum (SR) [1]. Also discussed elsewhere are changes in the intracellular pH, inhibitory effect on the ryanodine receptor channel activity [14], decreased intracellular inorganic phosphate [15], the enhancement of myofilament calcium responsiveness, and/or changes in cross bridge kinetics [16]. Since some of these potential mechanisms may occur simultaneously, we further investigate the inotropic mechanism of pyruvate and its relationship to the contractile properties of the heart. By examining a time-resolved picture of the positive inotropic effect as well as in parallel intracellular calcium handling under near physiological conditions in combination with assessment of pH and myofilament calcium responsiveness, we found that indirect enhancement of myofilament sensitivity plays a major role in the underlying mechanism for pyruvate’s inotropic effects.

## Materials and Methods

### Ethics Statement

All experiments were approved by the Animal Care and Use Committee of The Ohio State University and are in compliance with the laws of The United States of America and conform to the Guide for the Care and Use of Laboratory Animals published by the United States National Institutes of Health.

### Muscle Preparations

Male New Zealand White rabbits (2–3 months old ∼2 kg weight) were anesthetized by intravenous injection of sodium pentobarbital (60 mg/kg) following systemic heparinization with 5000 units/kg of Heparin. Hearts were rapidly excised and placed in Krebs-Henseleit buffer (K-H) composed of: 120 mM NaCl, 5 mM KCl, 2 mM MgSO_4_, 1 mM NaH_2_PO_4_, 20 mM NaHCO_3_, 0.25 mM Ca^2+^, and 10 mM glucose at a pH of 7.4. 20 mM BDM (2,3-butanedione 2-monoxime) was added to prevent cutting injury during dissection. From the right ventricular free wall, thin, uniform, non-branched, trabeculae were carefully dissected as previously described [17]. Muscle dimensions were carefully measured (average width, thickness and length were 0.18±0.02, 0.12±0.02, and 2.07±0.46 mm respectively, n = 22). By using only thin preparations, core hypoxia and ischemia were unlikely to occur and thus impair the results. Schouten and ter Keurs determined a critical diameter of 0.2 mm for room temperature experiments on rat [18], and studies by our lab at physiological temperature have shown that for the low frequencies used in our studies the thickness of the muscles used would not result in a hypoxic core in the muscles using this study protocol [18], [19], [20]. Muscles were placed into the experimental set-up, and superfused with the same K-H buffer as above at 37°C, without BDM, and with calcium raised to 1.5 mM. The preparation was allowed to stabilize for at least 30 minutes while electrically stimulated to contract isometrically at 2 Hz. The muscle was slowly stretched in small increments until increases in diastolic force equaled similar increases in active force development. It has been shown that, at this length, the length of the preparation is close to the *in vivo* end diastolic volume (representing a sarcomere length of ∼2.2 µm) [21].

### Intracellular Calcium and pH Measurements

Background auto-fluorescence was measured at excitation wavelength of 340 and 380 nm. The trabeculae were then iontophoretically loaded with bis-fura-2 (Texas Fluorescence Labs Inc, Austin, Texas.) as described previously [22], [23]. The iontophoretically loaded bis-fura-2 was allowed to spread via gap junctions throughout the preparation (at 22°C) until the photomultiplier output at baseline 380 nm excitation reached between 6 and 10 times over background. After loading of the dye was completed, the system was then switched back to 37°C, and data collection was started. The muscle was now continuously stimulated at 2 Hz while force and fluorescent emissions at 510 nm (excitation was alternated between 340 and 380 nm) were recorded. The emission signals were calibrated and converted to [Ca^2+^]_i_ by standard methods described by us [17] and others [22].

In a separate set of experiments, using a similar iontophoresis protocol as described above, the pH indicator BCECF was loaded into 5 muscle preparations. Light was then passed alternatively through 440 and 495 nm band-pass filters and fluorescence was monitored at 540 nm. From the subsequent calculation of the fluorescence ratio of the excitation wavelength of 495 and 440 nm, we obtained a qualitative estimate of intracellular pH. In these experimentsthe BCECF fluorescence ratio was not calibrated (since qualitative interpretation of the results should not be affected).The relative proton concentration was followed while pyruvate (10 mM) was applied as described above. No SR block was performed in this series of experiments.

### Pharmacological Inhibition of Sarcoplasmic Reticulum Function

To examine the effects of pyruvate independent of the effects on sarcoplasmic reticulum (SR) calcium handling, we performed a series of experiments in which pyruvate’s effect was assessed while the SR cycling of calcium was blocked. After loading of the fluorescent dye was accomplished and baseline twitch force and calcium transients were obtained, the inotropic response to pyruvate was assessed. The inotropic effect of pyruvate is dose dependent and has been shown to be present in rabbit ventricular trabeculae in concentrations that vary from 0.3 to 30 mM. At 5 to10 mM concentration there is a very pronounced effect that is easily reproducible [3], [16]. Pyruvate (10 mM) was initially applied for 20 minutes and thereafter completely washed out. At this point either 10µM of cyclopiazonic acid (CPA) and 1 µM of ryanodine (n = 6) or 100 nM of thapsigargin (n = 3) were added in order to block the SR [16], [24], [25]. Both strategies were chosen in order to ensure SR inhibition. After 30 minutes, and once the preparation was stabilized, an assessment of both force and calcium at the new baseline was performed. In the CPA+ryanodine preparations, rapid cooling contractures were performed before and after SR block to confirm that SR calcium handling was blocked. In all muscles, amplitude of the rapid cooling contracture were virtually zero (i.e. within the noise of the transducer), indicating a complete SR block, similar to previous studies [16], [26]. In both groups, pyruvate (10 mM) was again applied, and the response recorded in similar fashion under SR block conditions.

### Assessment of Myofilament Calcium Sensitivity

In a subset of experiments, after acquiring baseline values of both force and calcium transients, myofilament calcium sensitivity was measured at baseline and during the maximal pyruvate response. To obtain steady-state force-[Ca]_i_ relationships in intact muscle at body temperature, a potassium contracture protocol was used as described previously [17], [27]. Briefly, K^+^ contractures were elicited by switching the superfusion solution to a modified K-H solution containing high K^+^ (142 mM), 0 Na^+^, and 3 mM Ca^2+^. The other components of the K-H buffer remained unchanged. This high K^+^ solution was applied for 30 seconds and then washed out. During the developing steady-state contracture, intracellular calcium and force rise very slowly and are in near equilibrium. After washing out this high-K^+^ solution, the contraction relaxes, and the muscle returns to a normal twitching pattern with unaltered force or kinetics. From this data a force-calcium relationship can be constructed, and analyzed similar to the force-pCa curves typically obtained in skinned myocardium. Once the contractures and measurements were obtained under controlled conditions, pyruvate at 10 mM was added to the superfusate and for the next 20 minutes the experiments were repeated followed by washout.

### Skinned Fiber Experiments

To test the direct effects of pyruvate on the Ca^2+^ sensitivity of force development we employed chemically demembranated trabeculae as previously described [28], [29]. Rabbit trabeculae were harvested as described above. The buffers and solutions used for the force-pCa experiments are described in detail by Rall and by Moss [28], [30], [31]. Briefly, trabeculae were placed overnight at 4°C in relaxing solution containing 1% Triton X-100. This removes all membranes from the preparation including those from the SR, nuclei and inner and outer membranes of the mitochondria. The skinned trabeculae were mounted the next day between the arms of a high-speed length controller (model 322C, Aurora Scientific, Aurora, Ontario) and an isometric force transducer (model 403A, Aurora Scientific, Aurora, Ontario) with the sarcomere length adjusted to ∼2.2 µm, resulting in an average resting tension of 1.3±0.2 mN/mm^2^. The trabeculae were exposed to a range of randomized pCa solutions with the active force calculated as the total force minus the resting force (pCa 9.0). Maximum pCa activations (pCa 4.0) were measured at the beginning, middle and end of each Ca^2+^ titration and were used to normalize the developed force. Every pCa point was measured twice consecutively, either with or without the addition of 10 mM pyruvate (reversing the sequence for every other trabeculae). The force-pCa relationship were fit (using an iterative procedure) with a logistic sigmoid mathematically equivalent to the Hill equation, as previously described [32], [33]. All skinned trabeculae experiments were performed at 15°C.

### Data Acquisition and Analysis

Custom designed programs written in LabView (National Instruments, *Austin,* Texas) were utilized to collect and perform initial on line and off line data analysis. Data sets generated were then subjected to statistical analysis. Two-way and one-way ANOVA repeated measures with Tukey post-hoc testing or paired T-test were utilized where appropriate. Analysis was performed with the data analysis package provided with Kaleidagraph, (Synergy Software, Reading, PA). A p-value of <0.05 was considered significant. All data are depicted as mean ± SEM. For each protocol, only one preparation from each heart was included for statistical analysis.

## Results

### Inotropic Effects of Pyruvate

**Figure 1:**
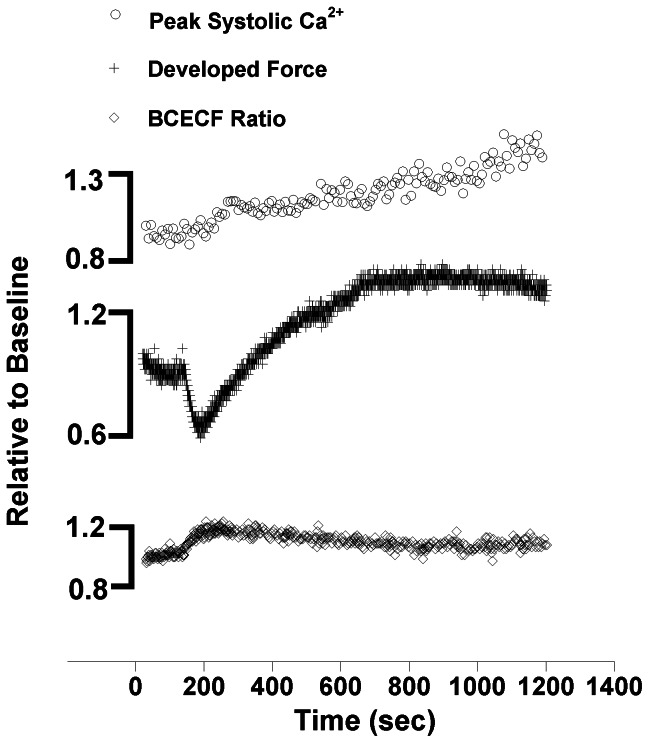
Single experiment recordings relative to baseline values: From top to bottom: peak bis-fura-2 fluorescence ratios (systolic calcium) showing a modest increase over time. The middle tracing displays the typical bimodal distribution of force development after superfusion of pyruvate. The bottom tracing is from a separate experiment where changes in pH were monitored with BCECF (an increase in the BCECF ratio corresponds to a decrease in [pH]_i_) showing the drop in [pH]_i_ concurrent with the drop in force. Time 0 indicates beginning of pyruvate application.

Typical twitch force recordings from a single experiment (Figure 1) show a transient decrease in active developed force between 2 to 3 minutes after application of 10 mM pyruvate (Time = 0), followed by a pronounced increase in developed force that stabilizes after about 10 minutes of pyruvate infusion. From the parallel assessed bis-fura-2 fluorescence, an increase in intracellular peak systolic calcium is observed as well. In other experiments (n = 5), using an identical protocol, we tracked pH via relative proton concentration during this same time course. From this data we can see that during the “dip” of force, the proton concentration transiently increases, reflecting a transient intracellular acidification, thereafter the ratio tends to return towards baseline values.(F_495/490_ ratio at baseline = 0.107; vs. “dip”  = 0.123 and vs. “peak” = 0.113, n = 5).

**Figure 2:**
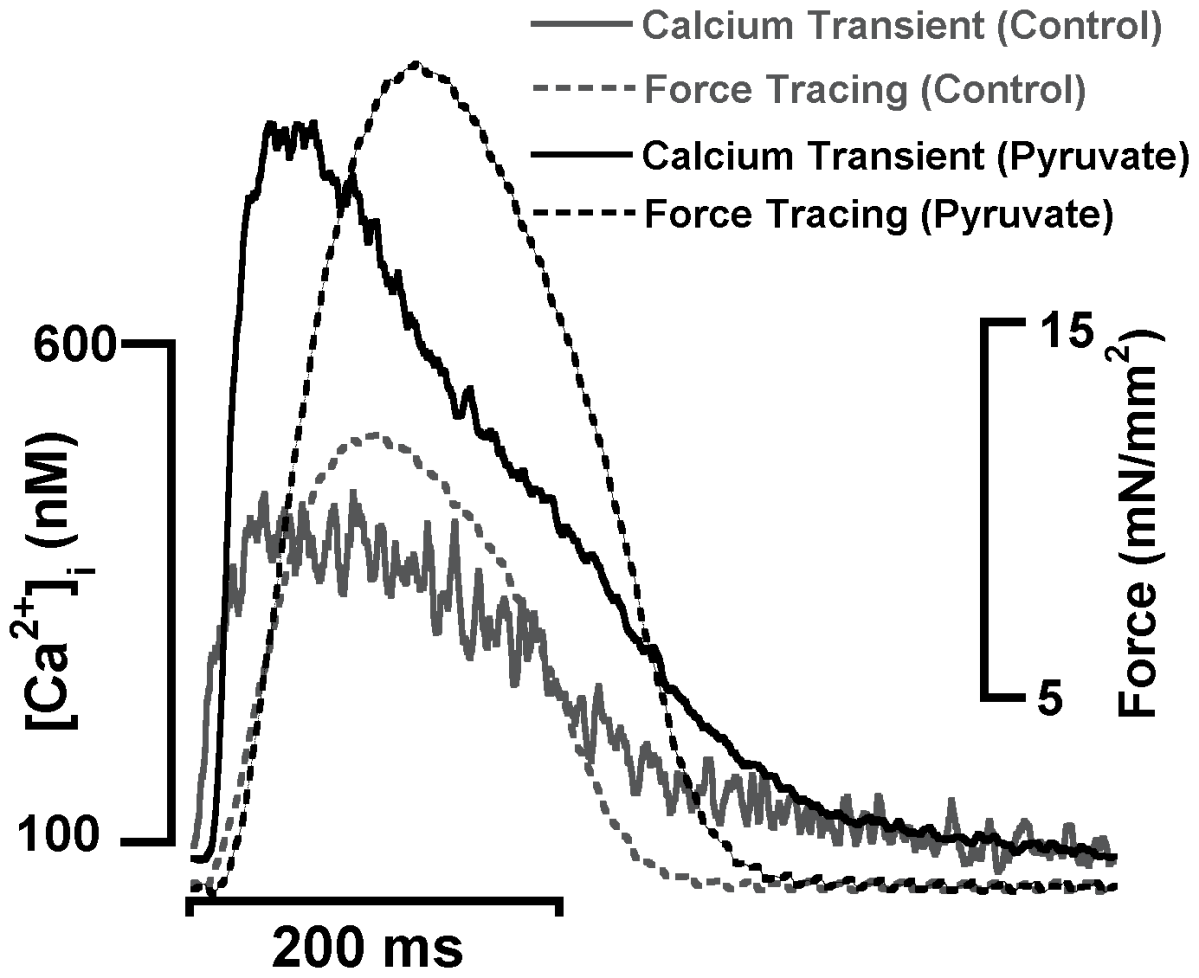
Snapshot of original transients at t = 20 minutes of developed force and intracellular calcium in rabbit trabeculae at 2 Hz during control versus pyruvate infusion. Data collected at 37°C. Pyruvate concentration was 10 mM.

Figure 2, displays the inotropic effects of pyruvate and its correlation with calcium transients from the same experiment. In this series pyruvate consistently increased force development to 146% of baseline values on average (range 112 to 182%, n = 9).

### Inotropic Effect is Preserved under Sarcoplasmic Reticulum Inhibition

**Figure 3:**
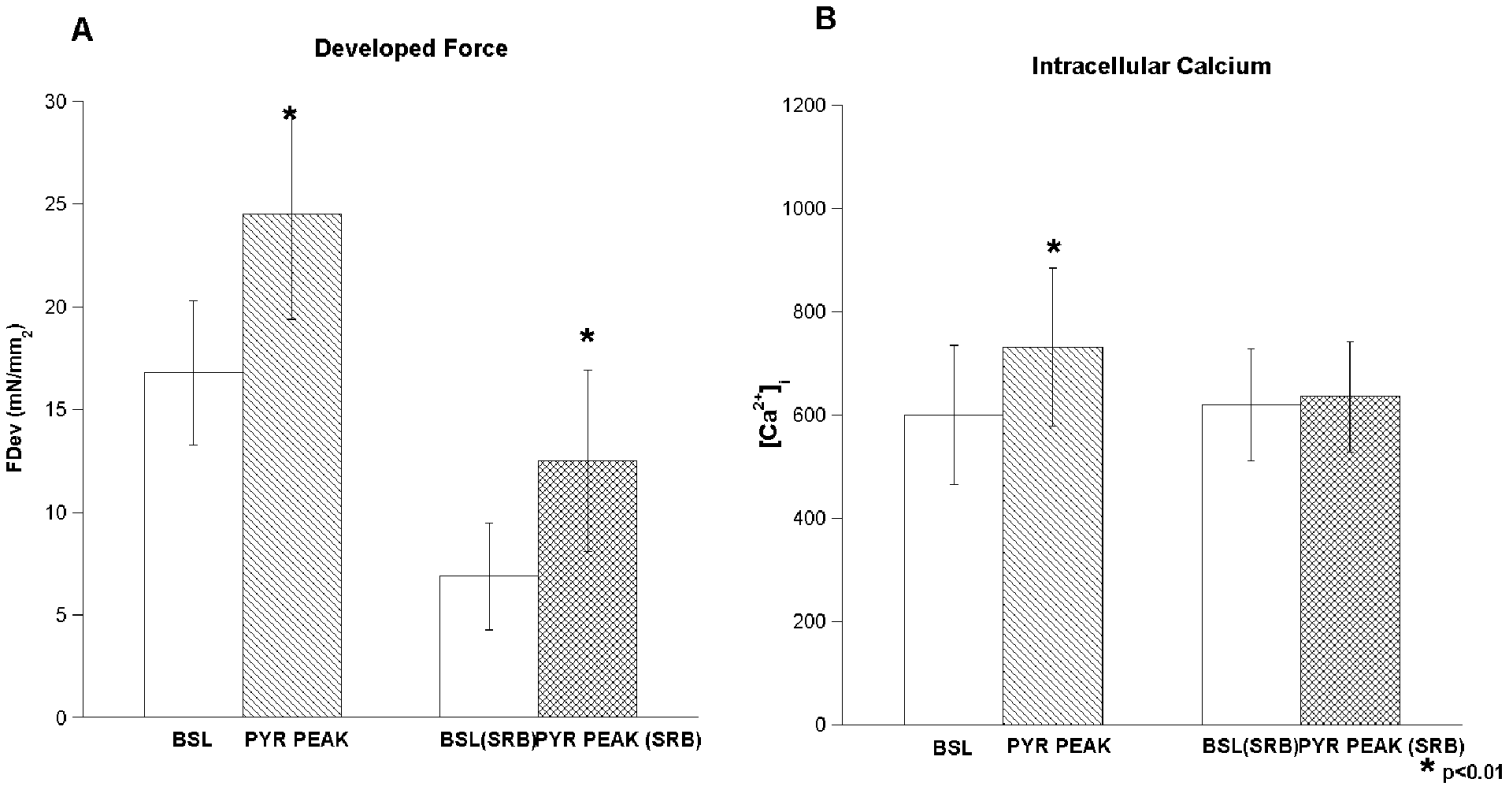
Panel A illustrates the increase in developed force compared to baseline after 10 mM of pyruvate to be similar in magnitude under both normal and blocked SR function. Panel B, the same experimental group before and after SR block now demonstrating the lack of significant change in calcium transient amplitude levels during pyruvate infusion. (Pyruvate 10 mM, 37°C) (p<0.01) n = 9.

Next, we tested the inotropic effect of pyruvate in the absence of functional SR calcium cycling. After pyruvate washout, the same trabeculae utilized above had the SR pharmacologically inhibited by a 30 minute exposure to the same KH solution with either cyclopyazonic acid and ryanodine (n = 6) or to thapsigargin alone (n = 3). Under SR block (indicated absence of any rapid cooling contracture) the baseline force decreased by an average of 59% from the control period (16.8±3.5 vs. 6.9±2.6 mN/mm^2^). Subsequently, and in continuous presence of SR block, pyruvate was again applied (10 mM), and the inotropic effect was again tested (Figure 3). We found that a similar magnitude of increase in force development is brought on by the infusion of pyruvate whether the SR is unblocked or blocked (16.8±3.5 to 24.5±5.1 vs. 6.9±2.6 to 12.5±4.4 mN/mm^2^, non-blocked vs. blocked SR respectively, p<0.001, n = 9). There was no appreciable difference in results whether thapsigargin or CPA+ryanodine was used to block the SR.

Importantly, during the non SR blocked (control) period an increase in intracellular calcium (as measured by peak systolic values) occurs during pyruvate infusion (from m, p<0.05) whereas during the SR block period, pyruvate infusion does not significantly change systolic calcium (620±108 to 635±107 nM, p = n.s.). Meanwhile, the increase in force still occurs to a large extent.

Table 1 summarizes the twitch parameters and cytosolic calcium kinetics during both the control and blocked SR periods. Baseline 1 refers to the initial pre pyruvate, pre SR block period. Baseline 2 is after the pyruvate washout and post SR block. Time to peak tension (TTP) and time from peak to 50% relaxation (RT_50_)are both significantly delayed after pyruvate infusion when the SR function is intact. Once the SR block is in place, there is still a trend towards delay in peak force development while the delay inRT_50_remains statistically significant compared to its respective baseline (pre pyruvate) period.

**Table 1:** Calcium Transient and Force Parameters.

parameter	TTP_(tension)_	RT_50_	RT_90_	TTP_(calcium)_
unit	ms	Ms	ms	ms
Control:				
Baseline	110±4	65±4	114±7	68±7
Pyruvate Peak	121±5^*^	78±4^*^	128±8^*^	57±5
SR Block:				
Baseline	182±12**	95±7**	181±14**	148±17**
Pyruvate Peak	210±17	107±7^*^	187±13	148±29

TTP _(tension)_ time to peak tension, RT _50_ time from peak tension to 50% relaxation, RT _90_ time from peak tension to 90% relaxation, TTP _(calcium)_ time to peak calcium. n = 9, *p≤0.05 (in comparison to the corresponding baseline). **p≤0.05 (control baseline versus SR block baseline). All values are average ± SEM.

### Myofilament Calcium Sensitivity is Significantly Increased by Pyruvate in the Intact Trabeculae but not in Skinned Fibers

**Figure 4:**
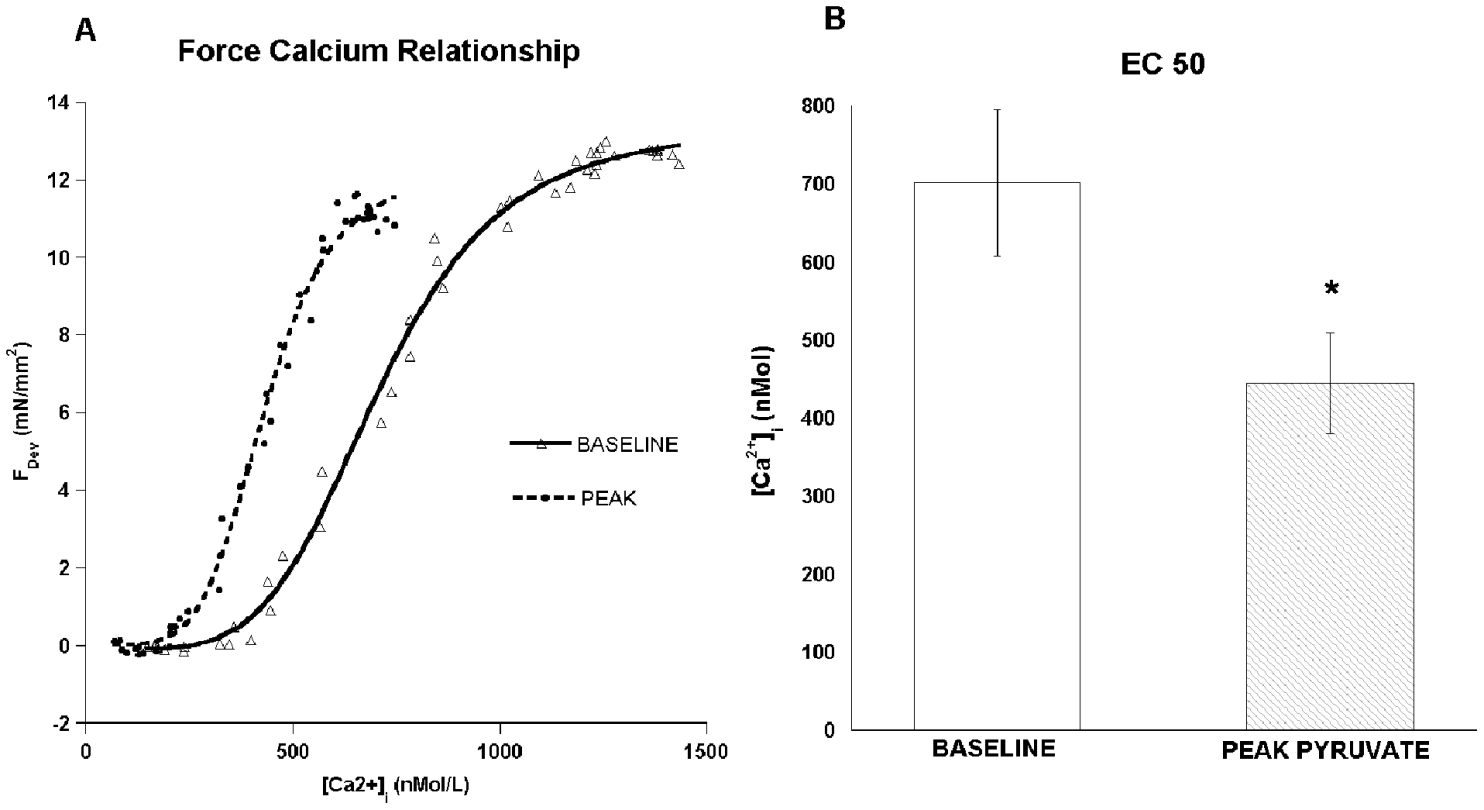
Potassium contracture experiment. A: Myofilament force-calcium curve. The myofilament calcium sensitivity curve shifts noticeably to the left denoting the increase in the calcium sensitivity at 20 minutes of pyruvate infusion (peak force development). Data points collected at baseline and at 20 minutes after the addition of 10 mM pyruvate, 37°C. B. Myofilament calcium sensitivity, expressed as EC 50 at baseline and at 20 minutes of exposure to 10 mM pyruvate (peak force development) demonstrates the sensitizing effect of pyruvate on the myofilaments (p<0.01, n = 6).

In figure 4A we show force and intracellular calcium data from potassium contracture experiments. The figure shows myofilament calcium sensitivity curves displaying the shift in sensitivity noticed from baseline (solid line), to 20 minutes of pyruvate infusion (peak force development, dashed line). These potassium contracture experiments were conducted after 20 minutes of baseline and at 20 minutes after the addition of pyruvate. The maximal developed force of these contraction was not significantly different in presence of absence of pyruvate, and was on average 27.2±4.9 mN/mm^2^ (n = 6). The shift to the left of this curve denotes the increase in the calcium sensitivity that occurs with the change in force during pyruvate infusion.

In figure 4B the aggregate myofilament calcium sensitivity, expressed as EC50 at baseline vs. 20 minutes of pyruvate infusion (peak force development) demonstrates the sensitizing effect of pyruvate on the myofilaments with a significant change in their values (701±94 vs. 445±65 nM, p<0.01, n = 6). Expressed as pCa50 values, pyruvate caused an increase from baseline of 6.17±0.06 to 6.37±0.06 (p<0.005, n = 6). The Hill coefficient increased from 3.9±0.4 to 7.2±0.9, (p<0.05, n = 6) from baseline to 20 minutes of pyruvate infusion.

**Figure 5:**
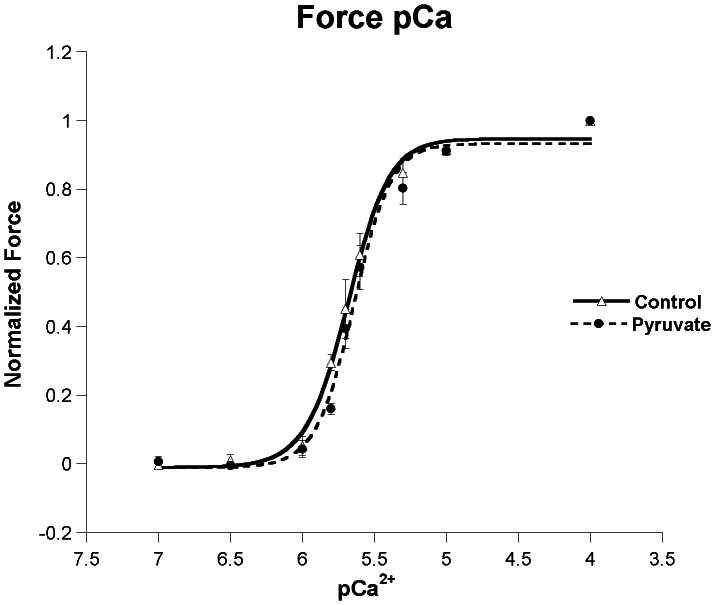
Skinned Fiber experiment: Force-pCa curve. The myofilament Force-pCa curve shifts minimally to the right denoting the non-significant change in the calcium sensitivity during pyruvate exposure (peak force development). Data points collected at baseline after the addition of 10 mM pyruvate, 37°C (p = n.s.), n = 5).

In skinned trabeculae the addition of pyruvate had no effect on the maximal developed tension of the skinned trabeculae (47.1±8.9 mN/mm^2^ in absence of pyruvate, and 46.9±10.3 mN/mm^2^ in presence of pyruvate, n = 8/group, p = n.s.) and as can be seen in figure 5, the equivalent parameters obtained from skinned fiber rabbit trabeculae caused a non-significant decrease in the Hill coefficient from 3.4±0.5 to 2.8±0.2 (p =  n.s., n = 5, figure 5), in contrast to the findings in the intact trabeculae. Similarly, EC50 did not change significantly (2.60±0.81 vs. 2.26±0.57 µM, control vs. pyruvate, p = n.s., n = 5), expressed as pCa values this was 5.60±0.08 vs. 5.66±0.06, p = n.s., n = 5).

## Discussion

Despite the fact that the inotropic actions of pyruvate are well documented, the processes underlying the inotropic effect are incompletely understood. Our findings strongly suggest that a change in apparent myofilament calcium sensitivity is a prominent physiological mechanism that underlies pyruvate’s enhancement of contractile force at the myocardial level.

It has been shown previously that pyruvate alters the calcium transient, and this finding has been used to explain pyruvate’s inotropic mechanism [16]. However, in most cases in which the inotropic mechanism involves an increase in the calcium transient amplitude, there is a concomitant acceleration of relaxation. Beta-adrenergic stimulation of the myocardium increases the force and developed calcium transient, but also accelerates the decline of the calcium transient and force development [34], [35]. Likewise, an increase in stimulation frequency increases the developed calcium transient and accelerates both calcium and force decline [27], [36]. In accordance with previous studies, we too observed that upon addition of pyruvate the calcium transient increases, but the decline of force does not increase, it actually is significantly slowed [3], [7], [8]. We demonstrated here that the increased SR calcium handling is not the primary underlying molecular mechanism for pyruvate’s inotropic effect. Moreover, even when the SR was entirely blocked from functioning, pyruvate continued to exert its inotropic effects that were quantitatively similar to those observed in non-blocked SR experiments. This leads us to conclude that there must be mechanisms downstream to SR calcium handling that are primarily responsible for pyruvate’s inotropic effect.

We have previously demonstrated that SR inhibition has a variable effect on the calcium transient that is both species- and frequency-dependent and that the balance between force and calcium is significantly different in intact trabeculae in larger animals (compared to rats) when studied under near physiological conditions [26]. In those experiments, inhibition of SR resulted in increased diastolic and peak calcium level as well as decreased developed force in both rats and rabbits. It was suggested that the myofilaments may have become further desensitized to calcium as a result of the increased in diastolic calcium concentration verified when the SR is inhibited. Species differences were further exacerbated at the higher frequencies (3–4 Hz in rabbit) where calcium transient amplitude decreased in rat but increased in rabbit [26], and can be explained by a loss of negative feed-back regulation of SR calcium release on the L-type calcium channel, resulting in increased L-type calcium entry.

Our focus thus turned next to evaluate the possibility that a change in myofilament calcium sensitivity might be at the basis of pyruvate’s inotropic effect. Using a recently developed potassium-based force-calcium assessment protocol [17], we observed a sensitization of the force-calcium relationship after 10 minutes of pyruvate exposure. This sensitization (left-ward shift) was very substantial, covering 0.2 pCa units, which is quantitatively similar to the magnitude of myofilament de-sensitization (rightward shift) typically observed with β-stimulation [37], [38]. In skinned fibers, we did not observe a significant sensitization, nor a change in apparent cooperativity of the myofilaments, leading us to conclude that pyruvate exerts its effects indirectly on myofilament calcium sensitivity. The differences we observed in maximal force between the skinned and intact fibers are due, at least in part, to different length (optimal versus 2.2 µm) and temperature (37 vs. 15 Celsius) that are known to impact force development [39], [40], [41], [42].

Unlike the impact of β-stimulation, which occurs in mere seconds, the inotropic impact of pyruvate takes many minutes to develop. In fact, before the force of contraction increases, it transiently decreases. Pyruvate gains entry to the cardiomyocyte via the monocarboxylate-proton symporter that co-transports pyruvate with a proton [43]. The initial dip in force characteristically present around 2 to 3 minutes after pyruvate perfusion is thus thought to be caused by a transient acidification of the intracellular pH [16]. This was followed by a gradual rise and the re-establishment of a new baseline pH. The latter finding, a reestablishment of pH, is disputed by Zima [14] as well as by Blatter and McGuigan and de Hemptinne et al. [44], [45] where sustained intracellular acidification was observed. Still, even in these previous reports, the reported changes in intracellular pH were deemed either insufficient to explain the magnitude and the differential effects of pyruvate on the contractile apparatus or the latter was not the subject of the investigation. Although not the focus of the current study, we conducted preliminary experiments to verify the relationship between the characteristic initial dip in force caused by pyruvate infusion and changes in intracellular pH. Similar to the previous study by Hasenfuss et al [16] and utilizing iontophoretically loaded BCECF, we verified the initial drop in twitch force to be coincident with a drop in intracellular pH as represented by the increase in F_495/490_ ratio. This was followed by a return of the ratio to (close to) baseline levels with no further alterations. From this we conclude the dip in pH is likely responsible for the dip in force development, but that the sustained inotropic effect of pyruvate is not mediated through altered proton concentration.

In this study we found that pyruvate exposure does augment calcium transients and that this augmentation likely plays some role in the genesis of the inotropic effect when the SR is intact. The increase in Ca^2+^ transients has been associated with increased SR calcium load and release. This finding is consistent with that of previous investigators [7], [16] and was observed in the experiments where the SR was not blocked. This could be explained by the sensitized myofilaments holding on longer to calcium ions, slowing their release, and possibly favoring re-uptake by the SR versus extrusion via NCX. In subsequent beats, the increased SR load would lead to an increase in the calcium transient amplitude. Thus, the increase in the calcium transient may be a secondary effect that is initiated by myofilament sensitization. Our results indicate that even in the absence of SR function, pyruvate will impose the majority of its inotropic effect and this effect is mediated through an increase in myofilament sensitivity. The increase in myofilament sensitivity would also explains the prolonged relaxation times in the presence of pyruvate.

Evidence from the skinned fiber experiments point away from any significant direct effect of pyruvate on the myofilaments themselves. Our data however does not allow us to rule out additional indirect non-myofilament effects to explain in part pyruvate’s inotropic properties such as its positive effects on phosphorylation potential and its known anti-oxidant effects.

At this point we can only speculate as to the exact molecular mechanism driving this modulation of myofilament sensitivity. Processes that are not immediate in onset (such as metabolic processes) are the most likely candidates. This would further explain the relatively slow development of the inotropic effect (minutes) seen with pyruvate, rather than much quicker effects such as observed after β-adrenergic stimulation, increased extracellular calcium, or changes in pre-load or frequency.

A decrease in inorganic phosphate has been shown to clearly occur with infusion of pyruvate [1], [12], [46], [47] and this decrease leads to an increase in calcium sensitivity of the myofilaments [48], [49]. In addition, the accompanying change in cytosolic phosphorylation potential with the resulting increase in free energy of ATP hydrolysis could also be contributing factors.

In summary, our results support the hypothesis that the main inotropic activity of pyruvate involves an indirect shift in myofilament calcium sensitivity. This can have important consequences for future development of therapeutic strategies for acute and chronic heart failure by circumventing the decreased effectiveness of current treatments that are believed to result in part from altered SR function in these pathophysiological entities.
